# Robotic versus laparoscopic and open surgery for endometrial cancer: a systematic review of randomized trials and pooled analysis of conversion rates

**DOI:** 10.1007/s11701-026-03625-w

**Published:** 2026-07-20

**Authors:** Emanuele De Angelis, Roberta Maria Arseni, Ilaria Cuccu, Innocenza Palaia, Giorgia Perniola, Ludovico Muzii, Emanuele Perrone, Giorgio Bogani, Evangelos Kontopantelis, Giuseppe Vizzielli, Violante Di Donato

**Affiliations:** 1 Department of Gynecological, Obstetrical and Urological Sciences, “Sapienza” University of Roma, Rome, 00161 Italy; 2 Department of Women Children and Public Health Sciences, Gynecologic Oncology Unit, Fondazione Policlinico Universitario Agostino Gemelli IRCCS, Rome, Italy; 3 Gynecological Oncology Unit, Fondazione IRCCS Istituto Nazionale dei Tumori, Milan, 20133 Italy; 4 Division of Informatics, Imaging and Data Sciences, University of Manchester, Greater Manchester, Manchester, UK; 5 Clinic of Obstetrics and Gynecology, Santa Maria della Misericordia University Hospital, Azienda Sanitaria Universitaria Friuli Centrale, Udine, Italy; 6 Department of Medicine, University of Udine, Udine, Italy

**Keywords:** “Endometrial cancer”, “Hysterectomy”, “Robotic”, “Laparoscopy”, “Laparotomy”, “Randomized”

## Abstract

To summarize randomized evidence assessing robotic surgery in relation to conventional laparoscopic and open abdominal approaches for endometrial cancer treatment, with particular attention to perioperative outcomes and conversion to open surgery. Randomized controlled trials evaluating robot-assisted surgical treatment of endometrial cancer were identified across major biomedical databases up to December 2025. Eligible studies compared the robotic approach against laparoscopic or open abdominal surgery. Quantitative pooling was undertaken only when outcome reporting was sufficiently consistent across studies. Eight randomized trials including 647 patients met the inclusion criteria. Overall, 322 patients underwent robotic surgery, 244 conventional laparoscopy, and 81 laparotomy. Most perioperative endpoints were reported heterogeneously, limiting formal pooling. Operative time varied across trials when robotics was compared with laparoscopy and was generally longer than laparotomy. Intraoperative blood loss and postoperative hospitalization did not show consistent differences between the two minimally invasive approaches. Compared with laparotomy, the robotic approach was linked to reduced postoperative stay. Conversion to laparotomy occurred less frequently after robotic surgery than after laparoscopy (0.7% vs. 8.4%; OR 0.17; *p* = .03). Complication reporting was inconsistent, although trials comparing robotics with laparotomy generally favored the robotic approach. Direct procedural costs were higher for robotics, whereas indirect costs favored robotics in the single study evaluating them. The robotic approach resulted in a lower need for open conversion compared with conventional laparoscopy. Other perioperative outcomes appeared broadly comparable between the two minimally invasive approaches, while comparisons with laparotomy suggested shorter hospital stay and fewer postoperative complications, although these findings should be interpreted cautiously because of the limited and heterogeneous randomized evidence.

**Supplementary Information:**

The online version contains supplementary material available at 10.1007/s11701-026-03625-w.

## Introduction

Endometrial cancer is the leading malignancy of the female genital tract globally, accounting for more than 417,000 incident cases and almost 100,000 deaths reported in 2020 [1, 2]. Because the disease is often detected at an early stage, surgical treatment plays a central role not only in oncologic management but also in determining perioperative morbidity and recovery. Selection of the surgical route is influenced by patient-related factors, including comorbidities and obesity, as well as by tumor characteristics, surgical complexity, and institutional expertise [3, 4].

For many women with endometrial cancer, minimally invasive approaches have gradually become preferred over open abdominal surgery because they may reduce operative trauma while preserving oncologic safety. Within this context, robotic platforms have been increasingly adopted in gynecologic oncology, offering three-dimensional visualization, articulated instruments, tremor filtration, and improved ergonomics. These technical features may be particularly relevant in patients with complex pelvic anatomy, high body mass index, or increased risk of conversion to laparotomy [5, 6].

Despite its widespread diffusion, the incremental benefit of robotic surgery over conventional laparoscopy remains debated. Much of the available evidence derives from retrospective cohorts or mixed-design reviews, which are vulnerable to selection bias, heterogeneity in surgical expertise, and differences in institutional practice. Randomized controlled trials are fewer, often small, and report perioperative outcomes inconsistently. Therefore, we conducted a systematic review restricted to randomized trials to assess short-term surgical results after robotic, laparoscopic, and open procedures for endometrial cancer, with a specific focus on the need for intraoperative conversion to laparotomy.

## Methods

### Search strategy

We searched the literature to identify randomized evidence evaluating robot-assisted procedures in patients with endometrial cancer. The review followed PRISMA guidance and was prospectively recorded in PROSPERO (CRD4202461183). The following sources were queried: PubMed/MEDLINE, EMBASE, Web of Science, Scopus, Cochrane Library, CINAHL, and ClinicalTrials.gov. The search used both indexed terms and free-text keywords covering endometrial cancer, hysterectomy, robotic surgery, laparoscopy, and randomization. The full search strategy was designed to capture studies comparing robotic hysterectomy with either conventional laparoscopy or laparotomy.

### Study selection

**Fig. 1:**
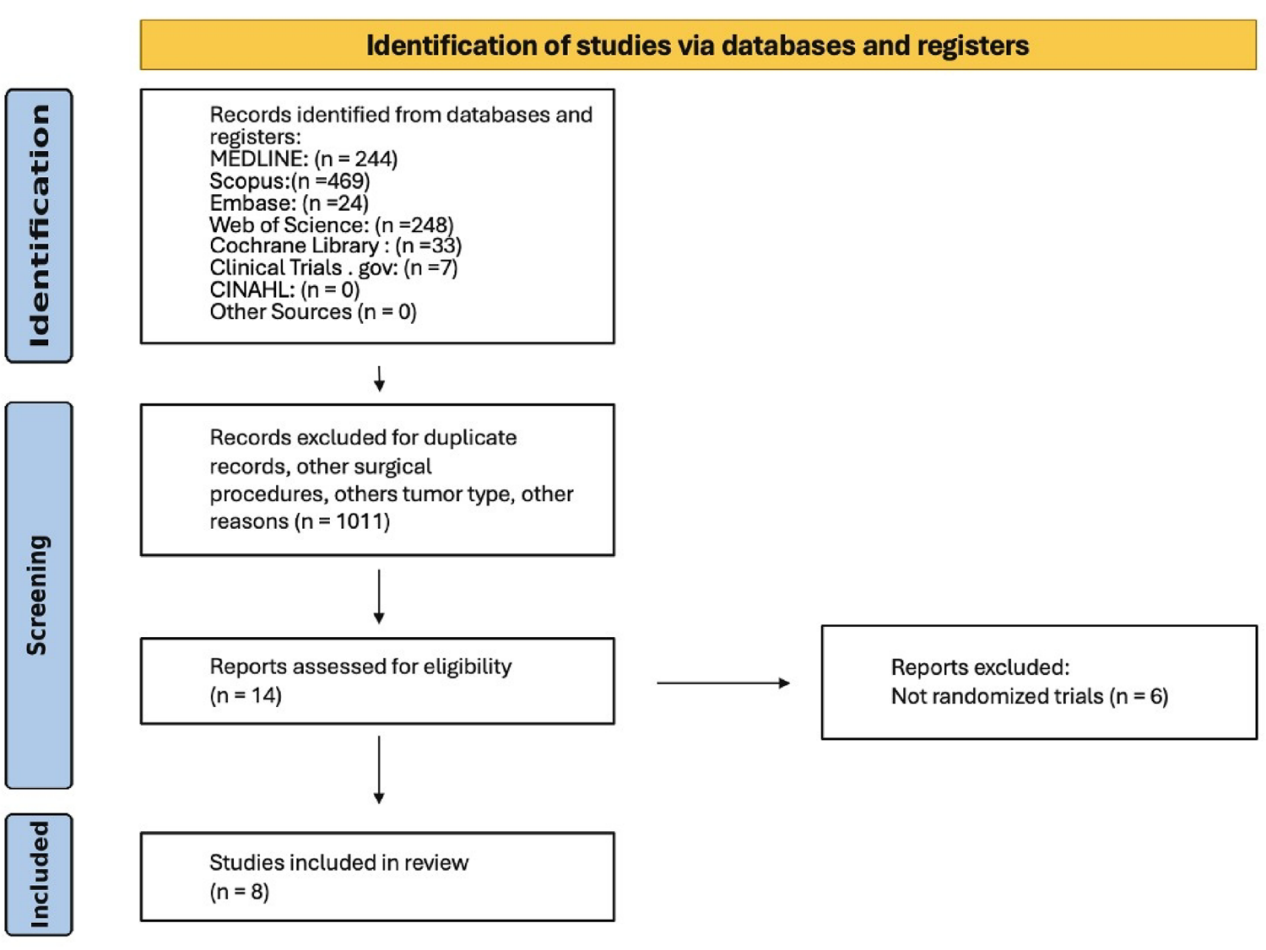
PRISHMA Flowchart

After duplicate removal, two reviewers (EDA and RMA) independently evaluated titles and abstracts. Articles considered potentially relevant by either reviewer underwent full-text assessment. Final eligibility was determined after discussion, and unresolved disagreements were adjudicated by a third reviewer (VDD). Figure 1 summarizes the complete screening and inclusion pathway.

### Eligibility criteria

We included only trials with randomized allocation involving women treated surgically for endometrial cancer and comparing a robotic approach with conventional laparoscopy or laparotomy. Eligibility was limited to English-language full-text publications. We excluded reports that involved benign gynecologic disease, cervical cancer, ovarian cancer, non-randomized designs, case reports, editorials, letters, narrative reviews, or conference-only material.

### Data extraction

For each included trial, two reviewers extracted study-level and outcome-level data using a predefined data collection framework. The dataset collected from each study comprised first author, year of publication, country of origin, trial design, cohort size, surgical comparator, patient numbers allocated to each arm, and reported perioperative endpoints. Prespecified endpoints included duration of surgery, intraoperative blood loss, hospital stay, conversion to laparotomy, perioperative complications, and cost-related measures.

### Data synthesis

Outcomes other than conversion to laparotomy were not pooled because of heterogeneity in definitions, reporting formats, summary measures, and available dispersion data and were synthesized descriptively; the specific reasons for each outcome are detailed in Supplementary Table S1.

Continuous endpoints including surgical duration, intraoperative bleeding, postoperative hospitalization, and economic measures were not pooled when studies reported non-comparable summary measures or lacked sufficient dispersion data. Dichotomous outcomes were collected as event numbers whenever available. Conversion to laparotomy was the only endpoint reported consistently enough to allow quantitative synthesis. For this outcome, robotic surgery was compared with conventional laparoscopy using odds ratios. Outcomes unsuitable for pooling were summarized narratively and displayed in comparative tables.

### Quality assessment and risk of bias

The reviewers independently assessed the risk of bias, using the Cochrane Risk of Bias tool for randomized trials (ROB-2 tool) [7]. We assessed bias in five domains (bias arising from the randomization process, bias due to deviations from intended interventions, bias due to missing outcome data, bias in measurement of the outcome, bias in selection of the reported result) plus overall risk of bias to classify each trial. Discrepancies in evaluating the risk of bias were resolved through discussions within the study team, leading to a final consensus before including the information. Potential overlap between study populations was carefully considered, particularly for studies conducted within the same research network or institution. Specifically, Salehi et al. 2017 [13] and Salehi et al. 2018 [12] both originated from the same trial, while Lundin et al. 2019 [15] and Lundin et al. 2020 [14] were derived from the same randomized trial population. Therefore, multiple reports from the same parent trial were treated as a single randomized population for the purposes of quantitative synthesis. Data were extracted from each publication only for distinct outcomes not reported in the corresponding primary report, in order to avoid double-counting patients.

### Outcomes

The review focused on perioperative and surgical outcomes. Endpoints included total operating room time, duration of the surgical procedure, intraoperative bleeding, postoperative hospitalization, need for conversion to open surgery, perioperative morbidity, and reported direct or indirect costs. When definitions differed across studies, outcomes were interpreted according to the terminology used by the original trial authors.

## Results

The Fig. 1 presents the study identification and selection pathway. The database and register searches retrieved a total of 1025 records. Following removal of duplicates and preliminary screening of titles and abstracts, 14 articles proceeded to full-text review. Six reports were removed because they were not randomized studies. Eight randomized studies, including 647 patients, were ultimately included in the review [8–15]. Four studies compared robotic surgery with conventional laparoscopy, whereas four compared robotic surgery with laparotomy (Table 1).

**Table 1:** Characteristics of included studies

First author	Year	Country	Study Design	Sample Size	Type of Surgery *N*
Minna M. Mäenpää [8]	2016	Finland	Randomized study	99	RH 50 LPS 49
Riikka-Liisa K. Vuorinen [9]	2017	Finland	Randomized study	101	RH 50 LPS 51
Alexandre Silva e Silva [10]	2018	Brazil	Randomized study	85	RH 42 LPS 43
Fabrice Narducci [11]	2020	France	Randomized study	200	RH 99 LPS 101
Sahar Salehi [12,13]	2017, 2018	Sweden	Randomized study	113	RH 56 LPTM 57
Evely Lundin [14,15]	2019, 2020	Sweden	Randomized study	49	RH 25 LPTM 24

RH: robotic surgery. LPS: laparoscopic surgery. LPTM: laparotomic surgery

**Fig. 2:**
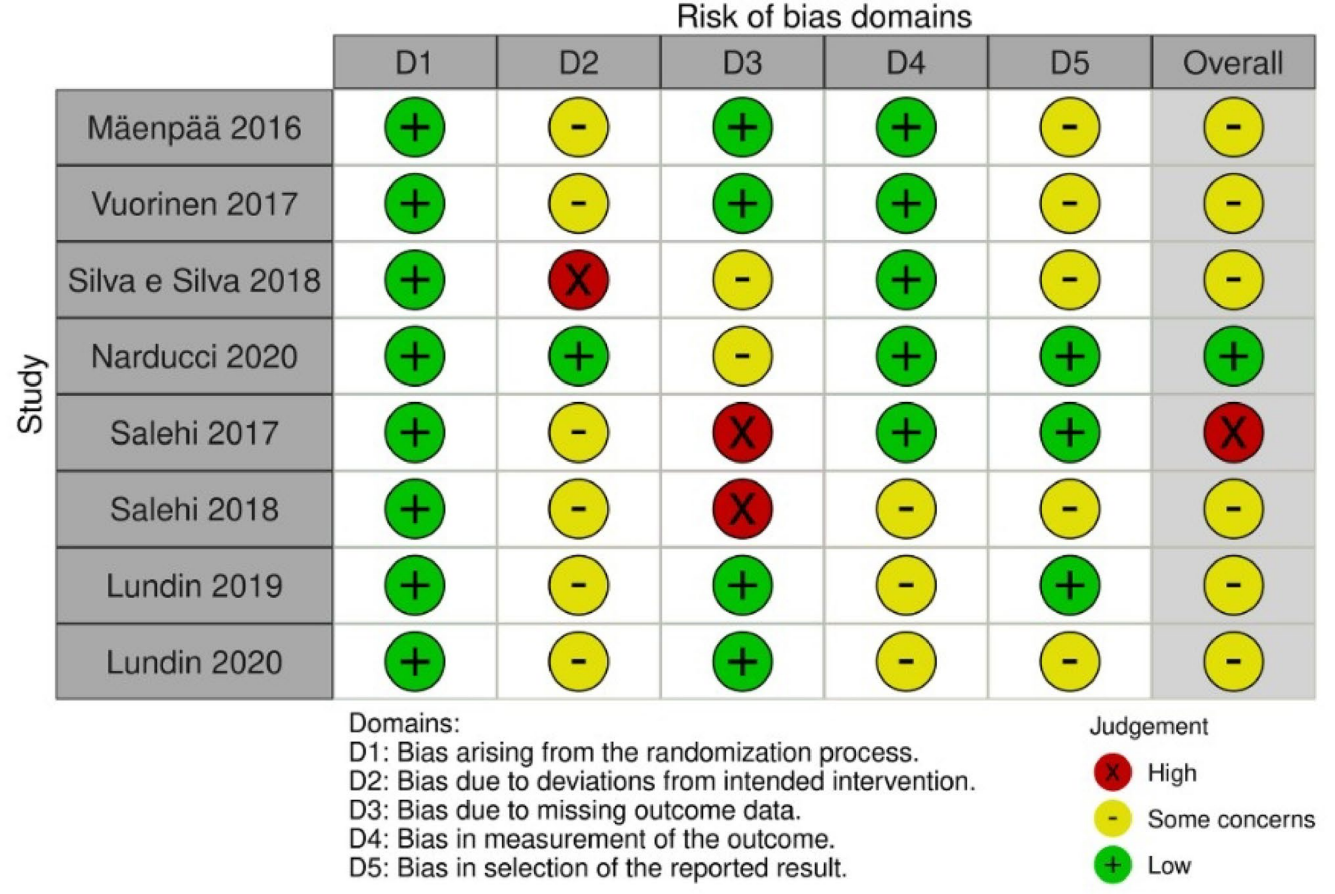
Risk of Bias Assessment

Figure 2 summarizes the risk-of-bias evaluation of the included trials.

Because reporting formats differed across trials, quantitative synthesis was not appropriate for most endpoints. Non-pooled outcomes are therefore presented descriptively and summarized in Table 2.

**Table 2:** Summary of Comparative Surgical Outcomes

Study (Year)	Type of Surgery *N*	Operative Time (min)	Operating Room Time (min)	Blood Loss (mL)	Length of Stay (days)	Conversion Rate (*N*)	Complications (*N*)	Total Cost (€ / USD)
Mäenpää et al., 2016 [8]	RH 50 LPS 49	139 vs. 170	197 vs. 228	50 vs. 50	1 vs. 2	0 vs. 5	IO: 0 vs. 4 PO: 18 vs10	NA
Vuorinen et al., 2017 [9]	RH 50 LPS 51	139 vs. 170	197 vs. 228	NA	1 vs. 2	0 vs. 5	NA	7.983 vs. 5.823 €
Silva et al., 2018 [10]	RH 42 LPS 43	NA	319.5 vs. 248	162 vs. 105.5	3 vs. 3	1 vs. 2	16 vs. 10	9.655 vs. 6.812 USD
Narducci et al., 2020 [11]	RH 99 LPS 101	184.5 vs. 132	NA	87.5 vs. 50	NA	NA	NA	NA
Salehi et al., 2018 [12]	RH 56 LPTM 57	229 vs. 183	NA	NA	NA	NA	4 vs. 5	NA
Salehi et al., 2017 [13]	RH 48 LPTM 48	233 vs. 187	NA	78 vs. 200	2 vs. 5	NA	11 vs. 16	14.505 vs. 16.807 €
Lundin et al., 2020 [14]	RH 25 LPTM 24	70 vs. 56	176 vs. 145	50 vs. 50	2 vs. 2	NA	NA	6.984 vs. 5,783 €
Lundin et al., 2019 [15]	RH 25 LPTM 24	70 vs. 56	NA	50 vs. 50	2 vs. 2	NA	2 vs. 5	NA

Among the 647 surgically treated patients included in the analysis, 322 (49.8%) underwent robotic surgery, 244 (37.7%) conventional laparoscopy, and 81 (12.5%) laparotomy. Histologic subtype was available for 429 patients: 320 (49.5% of the overall cohort) had endometrioid carcinoma and 109 (17.0%) had non-endometrioid disease, whereas histology was not reported in 218 cases (33.5%). Tumor grade was specified in 260 patients, including 211 (32.6%) with low- or intermediate-grade disease (G1–G2) and 49 (7.6%) with high-grade disease (G3); grade was unavailable in 387 patients (59.8%). FIGO stage was reported in 327 patients: 298 (46.0%) had stage I–II disease and 29 (4.5%) had stage III–IV disease, while staging information was missing for 320 patients (49.5%). Operative times showed inconsistent patterns between robotic and laparoscopic surgery: one study [10] showed a median “operating room time” increase in the robotic cohort compared to the laparoscopic one (319,5 vs. 248 min respectively, *p* < .001).

Conversely, two reports from the same randomized cohort found shorter operating room time with robotics than with laparoscopy (197 vs. 228 min, *p* < .001), as well as a shorter median operative time (139 vs. 170 min, *p* < .001) [8, 9]. In studies comparing robotics with laparotomy, the robotic approach generally required longer operative duration, including operative time of 233 versus 187 min (*p* < .001) [13], operative time of 70 versus 56 min (*p* = .048) [14], and operating room time of 176 versus 145 min (*p* < .001) [14]. Estimated intraoperative blood loss was available in milliliters for each included trial. In the comparison between robotic and laparoscopic surgery, one randomized controlled trial reported similar blood loss between groups [8], whereas two trials [10, 11] showed slightly greater blood loss in the robotic arm, without reaching statistical significance. When robotic surgery was compared with laparotomy, blood loss was comparable in two randomized controlled trials [14, 15] and significantly lower in one robotic cohort [13] (78 vs. 200 mL, respectively; *p* < .001).

Postoperative hospitalization showed no clear difference between robotic and laparoscopic groups, although two randomized controlled trials showed a non-significant trend favoring the robotic approach [8, 9]. In contrast, all comparisons with laparotomy reported shorter hospitalization after robotic surgery, with one study [13] demonstrating a clinically and statistically significant reduction in length of stay (2 vs. 5 days, respectively; *p* < .001).

**Fig. 3:**
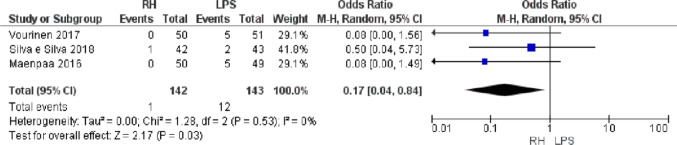
Forest plot of comparison: conversion of robotic vs. laparoscopy surgery

Robotic surgery was associated with a significantly lower risk of conversion to laparotomy. Across three randomized controlled trials [8–10], including 285 patients overall, conversion occurred in 0.7% of patients undergoing robotic surgery compared with 8.4% of those undergoing conventional laparoscopy (OR 0.17; *p* = .03), representing the only endpoint suitable for pooled analysis (Fig. 3).

Definitions and grading systems for complications varied substantially across studies. Severe complications were uncommon and did not differ significantly between robotic and laparoscopic arms. Minor complications were reported more frequently in robotic cohorts [8, 10]. In comparisons with laparotomy, two randomized controlled trials [12, 13] using Clavien–Dindo grading reported fewer minor and major postoperative complications in patients treated with robotic surgery.

Direct procedural costs were generally higher for robotic surgery, mainly because of instrumentation and personnel-related expenses. In the single trial evaluating indirect costs, measures such as sick leave and informal care were lower in the robotic arm [14]. No formal cost-effectiveness analysis was performed.

## Discussion

### Summary of main results

In this systematic review restricted to randomized trials, robotic surgery showed fewer conversions to laparotomy than conventional laparoscopy. Conversion was the only outcome reported with sufficient consistency to allow pooled analysis, showing a significant reduction in favor of the robotic approach. By contrast, duration of surgery, intraoperative blood loss, postoperative hospitalization, complications, and costs were reported with substantial variability across trials, limiting the possibility of broader quantitative synthesis. The overall pattern of evidence suggests that robotic surgery performs similarly to conventional laparoscopy for most short-term perioperative outcomes, while comparisons with laparotomy generally favor the robotic approach in terms of postoperative recovery and morbidity. However, these findings should be interpreted as qualitative signals rather than definitive comparative estimates, owing to the small randomized evidence base and the inconsistent reporting of outcomes across trials.

### Results in the context of published literature

The reduction in conversions is clinically significant given the morbidity associated with unplanned laparotomy. Robotic platforms likely reduce conversions by enhancing dexterity, stability, and visualization, particularly in anatomically challenging patients such as those with obesity or deep pelvic morphology. In technically complex cases, the improved visualization, instrument articulation, and ergonomic control provided by robotic systems may support more precise pelvic dissection and reduce the need to abandon the minimally invasive approach [16, 17]. These findings are consistent with a large retrospective analysis from a tertiary cancer center, which reported an overall conversion rate of only 2.4% for robotic gynecologic cancer surgery and as low as 0.5% for endometrial cancer specifically, with no association between conversion rates and BMI or age [18].

In obese patients with endometrial cancer, previous pooled evidence suggests a potential advantage of robotics in reducing conversion, particularly at higher BMI levels. In a meta-analysis limited to women with BMI ≥ 30 kg/m², conversion occurred in 6.5% of laparoscopic hysterectomies and 5.5% of robotic procedures; among patients with BMI ≥ 40 kg/m², the difference was more pronounced, with conversion rates of 7.0% and 3.8%, respectively. The same analysis also reported that Trendelenburg intolerance was a much more frequent cause of conversion during laparoscopy than robotics (31% vs. 6%), suggesting that the robotic approach may be better tolerated in morbidly obese patients [19]. A real-world population-based study from Poland confirmed these trends, reporting a 5.8% conversion rate in the laparoscopic cohort and no conversions in the robotic cohort among 640 patients operated in 2023 [16]. Compared with minimally invasive approaches, laparotomy requires a larger abdominal incision and wider tissue exposure, which may increase the risk of injury to adjacent organs such as bowel, bladder, and ureters, as well as wound complications and prolonged recovery [20]. Similar patterns have been reported in studies conducted in the United States, where a large propensity score-matched analysis of 35,224 patients from the Premier Healthcare Database demonstrated that the adoption of robotic surgery was associated with a reduction in laparotomy conversions and perioperative complications without an increase in short-term costs; robotic surgery facilitated the widespread diffusion of mini invasive approaches nationwide, with its use increasing from 9.5% to 56.8% during the study period [21]. However, this evidence should be interpreted with caution, as surgical training patterns differ significantly across healthcare systems. In the United States, many surgeons are now more extensively trained in robotic techniques than in conventional laparoscopy, which may partly account for the observed differences in surgical outcomes. The ACOG Committee Opinion suggests that the technical features of robotic platforms, particularly better visual depth and greater instrument control, may shorten the learning curve compared with standard laparoscopy; surgical efficiency appears to improve after roughly 20–30 procedures [22]. A recent analysis of the first 107 robotic procedures at a tertiary center confirmed rapid learnability, with a positive effect on the learning curve evident after approximately 20 procedures and a low conversion rate of 1.9% [23]. Therefore, these findings may not be directly generalizable to healthcare settings where laparoscopic training remains predominant. Robotics also outperforms laparotomy across multiple perioperative endpoints, including blood loss, complications, and length of stay. Although operative time tends to increase with robotics, this rarely translates into inferior outcomes, consistent with broader minimally invasive surgery literature [18, 19, 24–29]. A comprehensive network meta-analysis, including 37 studies and 6,558 participants, confirmed that robotic surgery is the most effective approach for reducing estimated blood loss, length of stay, transfusion rate, perioperative complications, and total complications compared with both laparoscopy and laparotomy [30]. The Cochrane systematic review, including nine RCTs and 3,944 women, reported no difference in survival outcomes, while confirming shorter hospital stays and lower rates of serious postoperative adverse events with laparoscopy compared with laparotomy [25]. These data provide the broader context within which the incremental benefits of robotics over conventional laparoscopy should be evaluated.

The randomized trials included in the present meta-analysis primarily focused on perioperative outcomes and did not specifically evaluate long-term oncologic endpoints such as overall survival, progression-free survival, or recurrence as primary outcomes. To date, one randomized controlled trial has provided long-term survival data comparing robotic-assisted with conventional laparoscopic surgery for endometrial cancer, finding a favorable overall survival in the robotic group (HR 0.39; 95% CI, 0.15–0.99; *P*=.047) but no difference in progression-free survival [31]. However, the small sample size and the restriction to low-grade histology limit the generalizability of this finding, and the authors themselves emphasized the need for larger randomized trials to confirm any potential survival benefit. In the absence of additional randomized evidence, the available data on long-term oncologic safety derive predominantly from retrospective cohort studies and meta-analyses. A large National Cancer Database analysis of 127,342 patients with stage I endometrial cancer found no difference in overall survival between robotic-assisted and traditional laparoscopic surgery (5-year OS: 91.7% vs. 91.4%; HR 1.00, 95% CI 0.96–1.04) [32]. Similarly, a multi-institutional retrospective study of 1,003 patients reported comparable 5-year disease-free survival (91.2% vs. 90.0%; *P*=.628) and overall survival (97.9% vs. 96.8%; *P*=.285) between robotic and laparoscopic cohorts [33]. Available pooled evidence on survival outcomes suggests that robotic surgery is oncologically safe in endometrial cancer, with long-term results comparable to conventional laparoscopy and potentially favorable compared with laparotomy [34]. Taken together, the current body of evidence supports the oncologic non-inferiority of robotic surgery compared with conventional laparoscopy, but the paucity of randomized long-term data remains a significant gap that future trials should prioritize.

The ergonomic advantages of robotic surgery for the operating surgeon represent an additional dimension that may influence the adoption and sustainability of this approach. The ROBOGYN-1004 randomized trial demonstrated that robotic-assisted laparoscopy significantly improved the perception of physical workload compared with standard laparoscopy, with lower perceived physical effort regardless of surgical complexity [35]. A paired cross-sectional study confirmed that robotic surgery is significantly less physically demanding than laparoscopy, with lower neck and shoulder muscle activity and lower ratings of perceived exertion [36]. These ergonomic benefits are particularly relevant for gynecologic oncology, where staging operations are lengthy procedures that can lead to surgeon fatigue and musculoskeletal complaints [37, 38]. According to the RCOG Scientific Impact Paper, robotic assistance may reduce the physical strain associated with surgery, as surgeons operating with robotic systems reported fewer musculoskeletal symptoms over both short- and long-term periods compared with non-robotic surgery [37]. A comparative meta-analysis of cost outcomes was not feasible due to substantial heterogeneity in cost reporting across trials. Nonetheless, direct costs, particularly instrumentation and personnel, were generally higher for robotic procedures, yet these differences were partly offset by shorter hospitalization and fewer perioperative complications. A prospective multicentre observational study from England found that, after adjusting for differences in morbidity, robotic surgery dominated conventional laparoscopy with lower costs and comparable quality-adjusted life years, although results were highly sensitive to the usage of robotic hardware [28]. In the context of endometrial cancer, the RCOG Scientific Impact Paper indicated that adoption of robotic surgery may be associated with better clinical outcomes for patients and potential economic benefits [37]. Importantly, no included study in our review conducted a formal cost-effectiveness analysis, and reported economic data were inconsistent, limiting definitive conclusions. Indirect costs were addressed in a single trial, in which robotic surgery was associated with reduced sick leave and informal care needs, suggesting potential long-term economic advantages. Overall, while current cost data remain heterogeneous, the balance of evidence indicates that robotic surgery may achieve favorable economic performance over time, particularly in complex cases and high-volume settings.

### Strengths and weaknesses

Strengths include the exclusive use of randomized evidence and adherence to PRISMA methodology, enabling comparison of the robotic approach with both standard laparoscopy and open abdominal surgery using randomized evidence. Limitations include heterogeneity in outcome definitions, use of medians instead of means, incomplete reporting of complications, and absence of survival or quality-of-life endpoints. Patients overlap across included publications represents an additional limitation that warrants explicit acknowledgement. Salehi et al. (2017) [13] and Salehi et al. (2018) [12] report different endpoints (perioperative outcomes and long-term quality of life, respectively) from the same Swedish RASHEC randomized cohort. Similarly, Lundin et al. (2019) [15] and Lundin et al. (2020) [14] report quality-of-life and cost-effectiveness outcomes from the same Swedish randomized trial of robotic versus abdominal hysterectomy, as evidenced by identical sample sizes and summary statistics. To avoid double-counting patients in the descriptive synthesis, each unique randomized cohort was counted once when reporting the total number of patients included in the review. The studies were used to extract distinct outcomes not previously reported. The small number of included trials (eight studies, 647 patients) limits statistical power and the ability to detect clinically meaningful differences in rare outcomes such as severe complications. Additionally, the included trials were conducted during a period of evolving surgical technique and platform technology, which may limit the applicability of earlier trial results to current practice.

### Implications for practice and future research

Robotics emerges as the minimally invasive approach with the lowest conversion rates, an important consideration for patients with obesity, prior surgeries, or limited pelvic exposure.

The ongoing RObese trial (NCT05974995), a phase III multi-institutional randomized study specifically designed to compare robotic versus laparoscopic surgery in obese patients with early endometrial cancer, with conversion rate as the primary endpoint, will provide important prospective evidence to guide surgical decision-making in this high-risk population [39]. Future randomized studies should adopt standardized perioperative outcome definitions and incorporate cost-effectiveness analyses that consider hospital expenditures, recovery-related costs, sick leave, and informal care. The Cochrane review on robot-assisted surgery in gynaecology emphasized that comprehensive economic analysis remains a critical unmet need, and that once clinical effectiveness is established, indirect factors such as surgeon well-being may become important cost considerations [40]. Adoption of robotic surgery should be guided by case complexity, institutional volume, and availability of dedicated training programs; centers implementing robotic programs should track conversion rates and perioperative outcomes prospectively to build local evidence bases.

## Conclusion

Robotic surgery was associated with a significantly lower conversion rate to laparotomy compared with conventional laparoscopy in randomized trials of endometrial cancer surgery. Comparisons with laparotomy suggested favorable perioperative outcomes with robotic surgery, particularly regarding blood loss, length of hospital stay, and postoperative complications; however, these findings were based on a limited number of trials and should be interpreted cautiously. The heterogeneity of outcome definitions and cost reporting across the available randomized evidence remains a critical limitation, underscoring the need for future trials adopting standardized endpoints, formal cost-effectiveness analyses, and patient-reported outcome measures. As robotic platforms continue to evolve and surgical training paradigms shift, prospective multicenter studies, such as the ongoing RObese trial, will be essential to define the optimal role of robotic surgery across different patient populations and healthcare systems.

## Supplementary Information

Below is the link to the electronic supplementary material.

Supplementary Material 1

## Data Availability

All data generated or analyzed during this study are included in this published article and its supplementary information files. No new individual patient-level data were generated. The data used for the analyses were extracted from previously published studies, as reported in the manuscript.
