# Supplementary material for: Robotic versus laparoscopic and open surgery for endometrial cancer: a systematic review of randomized trials and pooled analysis of conversion rates
Source: J Robot Surg. 2026 Jul 20;20(1):659. doi: 10.1007/s11701-026-03625-w (PMC13385253; doi:10.1007/s11701-026-03625-w)
Supplement: Supplementary file 1 — Supplementary Material 1 [file 11701_2026_3625_MOESM1_ESM.docx]

**Table S1.** Non-pooled Outcomes: Reporting and Rationale

|  | **Robotic vs Laparoscopy** | | | | **Robotic vs Laparotomy** | | | |  |
| --- | --- | --- | --- | --- | --- | --- | --- | --- | --- |
| **Outcome** | **Mäenpää 2016** | **Vuorinen 2017** | **Silva e Silva 2018** | **Narducci 2020** | **Salehi 2017** | **Salehi 2018** | **Lundin 2019** | **Lundin 2020** | **Main reason** |
| **Operative time/ operating room time** | Median; mean also reported | Median | Median | Median | Median | Mean | Median | Median | Different time definitions and summary measures |
| **Estimated blood loss** | Median | Not reported | Median | Median | Median | Not reported | Median | Median | Heterogeneous reporting and incomplete dispersion data |
| **Length of hospital stay** | Median | Median | Median | Not reported | Median | Not reported | Median | Median | Different definitions and reporting units |
| **Complications** | Intraoperative and postoperative; major complications reported separately | Not reported | Major/minor complications | Not reported | Clavien–Dindo complications | Postoperative complications grade >3 | Adverse events during stay and after discharge | Not reported | Variable definitions, grading systems, and time windows |
| **Costs** | Not reported | Median costs in EUR | Total/subcategory costs in USD | Not reported | Mean total costs in SEK/USD/EUR | Not reported | Not reported | Direct/indirect costs, QALYs, ICER in SEK | Different currencies, perspectives, cost categories, and time horizons |

**Note:** Due to substantial heterogeneity in outcome definitions, reporting formats, summary measures, and available dispersion data, quantitative synthesis was not feasible for these outcomes.
